# 21st International Conference on Emerging Infectious Diseases in the Pacific Rim

**DOI:** 10.3201/eid2511.191184

**Published:** 2019-11

**Authors:** Malla Rao, Robert Hall, Kristina Lu, Robin Mason, Gayle Bernabe, Patrick Brennan, Wolfgang Leitner, Christian Abnet, Kumiko Tsukui, Koji Yahara, Masahiro Yamamoto, Chie Nakajima, Yukari Totsuka

**Affiliations:** National Institutes of Health, Bethesda, Maryland, USA (M. Rao, R. Hall, K. Lu, R. Mason, G. Bernabe, W. Leitner, C. Abnet);; Colorado State University, Fort Collins, Colorado, USA (P. Brennan);; National Institute of Infectious Diseases, Tokyo, Japan (K. Tsukui, K. Yahara);; Osaka University, Osaka, Japan (M. Yamamoto);; Hokkaido University, Sapporo, Japan (C. Nakajima); National Cancer Center, Tokyo (Y. Totsuka)

**Keywords:** Conference, emerging infectious diseases, Pacific Rim, United States, Japan

The U.S.–Japan Cooperative Medical Sciences Program (USJCMSP) was established in 1965 by Prime Minister Eisaku Sato of Japan and President Lyndon B. Johnson of the United States to strengthen Japan’s research capacity and address public health concerns in the Asia-Pacific region (https://www.amed.go.jp/en/program/list/03/01/007.html). USJCMSP currently has 9 joint panels of scientific experts overseeing research in acute respiratory infections; AIDS; cholera and other bacterial enteric infections; hepatitis; nutrition and metabolism; cancer; parasitic diseases; mycobacterial diseases; and viral diseases, as well as a cross-cutting immunology board.

In 1996, USJCMSP pioneered the International Conference on Emerging Infectious Diseases of the Pacific Rim. The conferences, held in countries in the Asia-Pacific region (Table), provide a venue for panel meetings, discussions on infectious disease research, and opportunities for collaboration.

**Table:** Topics and locations of the US-Japan Cooperative Medical Science Program International Conference on Emerging Infectious Diseases in the Pacific Rim, 1996–2019*

Date	Venue	Topics
July 1996	Kyoto, Japan	Introduction to the conference initiative
March 1997	Bangkok, Thailand	Dengue, EHEC, drug-resistant bacteria
March 1998	Bali, Indonesia	Tuberculosis, malaria, hepatitis, carcinogenesis
March 1999	Bangkok, Thailand	Parasitic infections and influenza
January 2000	Chennai, India	Tuberculosis, leprosy, and AIDS
January 2001	Manila, Philippines	Bacterial diarrhea and viral zoonosis
October–November 2002	Shanghai, China	Acute respiratory infections and parasitic zoonosis
December 2003	Dhaka, Bangladesh	Cholera and bacterial enteric infections; AIDS
December 2004	Kyoto, Japan	Influenza; 40^th^ anniversary of USJCMSP
November 2005	Hanoi, Vietnam	AIDS; tuberculosis
November 2006	Singapore	Avian influenza
December 2007	Haikou, Hainan, China	Antimicrobial resistance in respiratory infections
April 2009	Kolkata, India	Enteric diseases
October 2010	Penang, Malaysia	Next-generation diagnostics of infectious diseases
March 2013	Singapore	Viral vaccines and protective immunity
February 2014	Dhaka, Bangladesh	Antimicrobial drug resistance in bacterial and parasitic diseases
January 2015	Taipei, Taiwan	Emerging viral diseases: recovery and control
January 2016	Bethesda, MD, USA	Emerging research technologies and innovations; 50^th^ anniversary of USJCMSP
February 2017	Seoul, South Korea	Antimicrobial drug resistance in bacterial and parasitic diseases
January 2018	Shenzhen, China	Pathogenesis and protective immunity of viral diseases
February 2019	Hanoi, Vietnam	Bacterial and parasitic diseases
*EHEC, enterohemorrhagic *Escherichia coli*; USJCMSP, US-Japan Cooperative Medical Science Program.		

The 21st conference, hosted by the Ministry of Health of Vietnam and held in Hanoi during February 26–March 1, 2019, focused on bacterial and parasitic diseases. The conference program included 5 plenary sessions: One Health; genomics; antimicrobial resistance (AMR); technologies and heterogeneity/single cell; and the microbiome, immunity, and cancer. The conference program and other materials are available on the conference website (http://www.cvent.com/events/u-s-japan-cooperative-medical-sciences-program-usjcmsp-21st-international-conference-on-emerging-inf/event-summary-a5489b8e72fb476ca8ff229650f812e3.aspx).

One Health takes a holistic view of hosts, vectors, zoonotic reservoirs, and the ecosystem to study emerging diseases and disease transmission. In the Asia-Pacific region, viruses that cause human illnesses, such as influenza and Nipah virus, harbor in birds or bats, move into zoonotic reservoirs, and adapt and mutate in intermediate hosts before infecting humans. Presenters at this year’s conference described an initiative in Bhutan that created a One Health South Asian network, extending to Afghanistan, Nepal, and Bangladesh. The initiative enables training in epidemiology, animal health, molecular methods, and economic analyses applicable to tracking brucellosis, rabies, Q fever, toxoplasmosis, Japanese encephalitis virus, and avian influenza. Other One Health topics included tuberculosis transmission from humans to elephants in Nepal; deer, rhinoceroses, and blue bulls transmitting *Mycobacterium orygis*; Rhesus monkeys dying of tuberculosis in Bangladesh; genetically distinct *Bacillus anthracis* in humans in Vietnam; and antimicrobial-resistant strains of *Listeria monocytogenes* isolated from carrots, eggplants, and other crops in Malaysia.

The session on genomics covered its application to investigate genetic diversity, epidemiologic complexity, pandemic outbreaks, pathogen resistance, and host–pathogen interactions in the context of malaria, tuberculosis, and cholera. *Plasmodium vivax* accounts for >80% of the global burden of malaria in the Asia-Pacific region. Presenters described how *P. vivax* in Papua New Guinea exhibits greater genetic diversity and epidemiologic complexity and resistance to control than *P. falciparum*. Other presenters discussed genomic analysis of *P. simium,* which caused an outbreak of human malaria in the Atlantic Forest of Rio de Janeiro. They found *P. simium* from the outbreak has a close genetic and morphological resemblance to *P. vivax*, and their phylogenetic analysis juxtaposed the 2 species. Their analysis suggests that *P. vivax* originated in great apes in Africa before infecting humans and that after 1492, human hosts transported *P. vivax* to South America, where it adapted to New World monkeys and further evolved into infectious *P. simium*.

Another genomic study explored the current >50-year cholera pandemic, the cause of which is a limited number of closely-related clades of *Vibrio cholera.* Genomic evidence points to the pathogen’s evolution in dense human populations of the Ganges Delta, with seeding introductions of cholera to other countries and continents.

The session on AMR focused on drug resistance in different community settings, microbial mechanisms to mediate AMR, and coordinated actions to minimize the emergence and spread of AMR. Presenters described infection control measures in hospitals in Vietnam, notably for carbapenem-resistant *Enterobacteriaceae* (CRE), which show strong correlations between CRE colonization, hospital-acquired infections, duration of treatment, and fatality. 

In Japan, prevalent AMR infections include enterohemorrhagic *Escherichia coli* (EHEC) and healthcare-associated infections, such as CRE, multidrug resistant *Acinetobacter* sp., and vancomycin-resistant *Enterobacteriace*. Presenters described the use of multiple-locus variable number tandem repeat analysis to detect diffuse outbreaks and prevent disease transmission by identifying an identical genotype of EHEC.

In the Greater Mekong Subregion, artemisinin-resistant malaria is widespread and associated with evolving polymorphisms in the *P. falciparum* Kelch propeller K13 gene. *P. vivax* is the predominant parasite and a presenter described how resistance to chloroquine might involve novel mutations in the *Pvmdr1* gene. Another AMR presentation described the continuing challenges of cholera in Kenya, where *V. cholera* demonstrates resistance against β-lactam and disease hotspots are expanding to refugee camps despite improvements in sanitation. 

The technologies and heterogeneity/single cell session explored recent technological advances in tracking disease progression, evaluating organisms responsible for human infectious diseases, and characterizing stages of disease manifestations. A talk on *Helicobacter pylori* covered how it colonizes and limits repair at gastric damage sites via chemotactic motility. The presenters are investigating mutations in chemokine receptors, which could help define the role of chemotaxis in *H. pylori* pathogenesis. Another presentation discussed RNA interference as a promising tool against mosquitoes and for mosquitoborne disease control. A third presenter described the use of CRISPR-based functional genomics for identifying new drug targets for tuberculosis and how progression of *Mycobacterium tuberculosis* infections can be tracked by combining positron emission tomography and computed tomography with immunology and microbiology in in vivo models. A final presenter discussed the use of whole genome sequencing to reconstruct the migration and expansion of human infectious diseases by using phylogenetic reconstruction of *Schistosomiasis japonica* to demonstrate its evolution through rice planting, agricultural development, human movement, and population increases.

The session on the microbiome, immunity, and cancer included a metabologenomic study demonstrating that lactate derived from dietary fiber fermentation by lactic acid bacteria accelerates colonic epithelial cell turnover and development of aberrant crypt foci after carcinogen treatment. Another speaker described how the gut ecosystem, formed by microbiota, has a profound influence on human physiology, immunology, and nutrition, and imbalance in its structure could increase risk for colorectal cancers. In a human study described during this session, the fecal microbiome and its metabolite profile shifted from early stage polyps to colorectal carcinoma, and researchers found specific fecal microbes and metabolites correlated with the stage of colorectal carcinoma. The research suggests gut microbiota–derived metabolites could factor into our understanding of tumorigenesis and provide information for early detection and prevention strategies. Another speaker’s research found that tuberculosis exposure is associated with microbiome changes in patients in Haiti and induction of an early innate immune response. 

